# Ocular Delivery of Compacted DNA-Nanoparticles Does Not Elicit Toxicity in the Mouse Retina

**DOI:** 10.1371/journal.pone.0007410

**Published:** 2009-10-12

**Authors:** Xi-Qin Ding, Alexander B. Quiambao, J. Browning Fitzgerald, Mark J. Cooper, Shannon M. Conley, Muna I. Naash

**Affiliations:** 1 The Department of Cell Biology, University of Oklahoma Health Sciences Center, Oklahoma City, Oklahoma, United States of America; 2 Copernicus Therapeutics, Inc., Cleveland, Ohio, United States of America; University of Florida, United States of America

## Abstract

Subretinal delivery of polyethylene glycol-substituted lysine peptide (CK30PEG)-compacted DNA nanoparticles results in efficient gene expression in retinal cells. This work evaluates the ocular safety of compacted DNA nanoparticles. CK30PEG-compacted nanoparticles containing an EGFP expression plasmid were subretinally injected in adult mice (1 µl at 0.3, 1.0 and 3.0 µg/µl). Retinas were examined for signs of inflammation at 1, 2, 4 and 7 days post-injection. Neither infiltration of polymorphonuclear neutrophils or lymphocytes was detected in retinas. In addition, elevation of macrophage marker F4/80 or myeloid marker myeloperoxidase was not detected in the injected eyes. The chemokine KC mRNA increased 3–4 fold in eyes injected with either nanoparticles or saline at 1 day post-injection, but returned to control levels at 2 days post-injection. No elevation of KC protein was observed in these mice. The monocyte chemotactic protein-1, increased 3–4 fold at 1 day post-injection for both nanoparticle and saline injected eyes, but also returned to control levels at 2 days. No elevations of tumor necrosis factor alpha mRNA or protein were detected. These investigations show no signs of local inflammatory responses associated with subretinal injection of compacted DNA nanoparticles, indicating that the retina may be a suitable target for clinical nanoparticle-based interventions.

## Introduction

Inherited retinal degenerative diseases are a major cause of blindness worldwide. Defects in a large number of genes can cause retinal degenerative disorders (http://www.sph.uth.tmc.edu/RetNet/disease.htm), but there are currently no effective treatments for the diseases. Due to the monogenic nature of many inherited retinal diseases, gene replacement/correction therapy is one of the most promising treatment options. Viral-meditated gene delivery and therapy has been successful in various animal models and it is currently generating promising results in clinic trials [1], [2]. For example, restoration of retinal function by viral-mediated gene delivery was documented in canine (*RPE65* ^−/−^ dog) [3], [4] and mouse models of Leber Congenital Amaurosis, in mouse models of retinitis pigmentosa (*rd* mice) [5], and in mouse models of complete achromatopsia (*Gnat2* ^−/−^ mice) [6]. Human patients have shown improvement after delivery of ciliary neurotrophic factor by intraocular implant [7] and after viral delivery of RPE65 [1], [2], [8]. In spite of these successes, safety concerns have increasingly surfaced for viral vector-mediated gene transfer, and some trials have resulted in oncogenesis or even mortality [9], [10] (albeit non-ocular trials). Additionally, broad application of traditional viral gene delivery can be hindered by limitations in the size of the expression cassette to be transferred, host immunity to repeat infections, and the possibility of insertional mutagenesis [10], [11]. A recent report showing that retinal delivery of the viral vectors has resulted in viral vector DNA in the brain [12] would raise a concern regarding use of viral vectors in the eye. The widespread development of viral vectors for clinical use also faces practical and economical challenges. There are more than 140 genes that cause retinal degenerative disorders, many of which contain multiple single disease causing mutations. For gene replacement therapy, each additional gene requires the development of a new vector (viral or non-viral). Given the time and expense involved in generating and testing viruses, the development of a less expensive alternative such as nanoparticles is beneficial.

Recently a variety of non-viral gene delivery systems have been developed to supplement the available viral treatment options. One such approach is the use of DNA condensed with polycationic polymers. Compacted DNA nanoparticles with different formulations have been shown to be efficient in gene transfer to a number of tissues [13], [14], [15]. One particularly successful formulation consists of a single molecule of plasmid DNA compacted with polyethylene glycol (PEG)-substituted polylysine (CK30PEG) [16]. These nanoparticles are effective in delivering genes to dividing and post-mitotic cells and have a plasmid capacity of at least 20 kbp [16], [17]. They efficiently deliver DNA to the murine lung with minimal toxicity and animals can be repeatedly dosed without decrement in biologic activity [15], [18]. These nanoparticles have been used in a phase I/II clinical trials to deliver the cystic fibrosis transmembrane regulator (CFTR) gene to cystic fibrosis (CF) patients [19] and are also being developed for treatment of genetic brain diseases [20]. Recently, we have demonstrated successful ocular delivery of these CK30PEG nanoparticles (containing a CMV-EGFP vector, pZEOGFP5.1) [14]. The nanoparticles were targeted to different tissues within the eye by varying the site of injection; almost all cell types of the eye were capable of being transfected. Of particular interest for the treatment of inherited retinal degenerations, subretinal delivery of these nanoparticles transfected nearly all of the photoreceptor population and did not cause irreversible defects in retinal function (as measured by electroretinogram (ERG)) [14]. Our most recent studies have demonstrated that these nanoparticles can be used to provide partial structural and functional rescue of a retinitis pigmentosa disease phenotype after subretinal delivery to the diseased mouse eye [21].

These encouraging results make further analysis of the safety and toxicity of these nanoparticles critical. This step has not been undertaken and is necessary for the progress of this technology and the development of novel nanoparticle based treatments. While the eye has traditionally been considered an immune privileged tissue, it does exhibit immune reactivity under certain stresses and is prone to environmentally-induced alterations. Although we have previously shown that retinal function (ERG recordings) was not affected by delivery of the nanoparticles [14], no effort has been made to learn how the local tissues respond to the nanoparticles and whether there is a local toxic or inflammatory response. To that end, the purpose of this work was to evaluate any retinal inflammatory or cellular toxic responses to nanoparticle administration. Here we report that subretinal delivery of DNA nanoparticles does not induce inflammatory infiltrates or significant levels of cytokines. These findings suggest that ocular delivery of DNA nanoparticles is a safe approach for future clinical testing for the treatment of inherited retinal degeneration.

## Results

### Expression of Transgene

**Figure 1:**
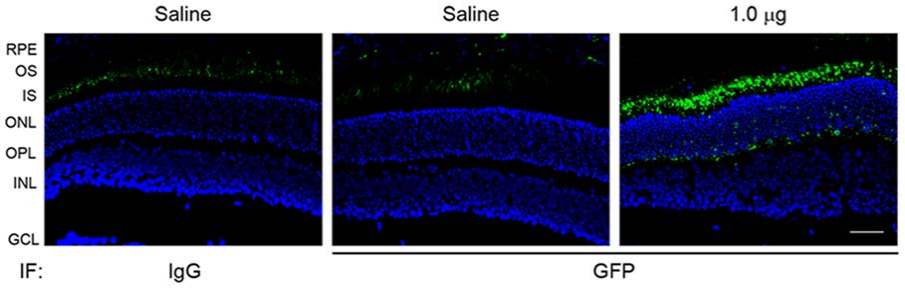
Immunofluorescence detection of EGFP expression in mouse retinas following subretinal delivery of compacted DNA nanoparticles or saline. Immunofluorescence labeling using anti-GFP was performed on paraffin-embedded sections. Shown are representative images of EGFP immunolabeling on the retinal sections of eyes that had been injected with 1.0 µg nanoparticles or with saline at PI-2. Immunoreactivity was detected in the IS, ONL, and OPL layers. Retinal sections were also incubated with normal IgG as negative control. RPE, retinal pigment epithelium; OS, outer segment; ONL, outer nuclear layer; OPL, outer plexiform layer; INL, inner nuclear layer; GCL, ganglion cell layer. Scale bar, 50 µm.

Retinal expression of EGFP following subretinal delivery of CMV-EGFP (CK30PEG pZEOGFP5.1) nanoparticles was examined by immunofluorescence labeling using anti-GFP antibody. Figure 1 shows representative images of EGFP expression in the retinal sections at 2 days post-injection (PI-2). EGFP immunoreactivity was detected in inner segments (IS) and in the outer nuclear layer (ONL). No immunoreactivity was detected in saline-injected eyes or in retinal sections that had been incubated with normal IgG (Figure 1). Consistent with previous experiments using this vector [14], transgene expression was not detected at PI-4 or PI-7. The short duration of transgene expression is likely related to the rapid silencing of the CMV promoter. We have observed much longer duration of transgene expression after a single delivery of these nanoparticles (up to one year) when other promoters such as the interphotoreceptor retinoid binding protein promoter or the chicken beta-actin promoter are used.

### Infiltration and Activation of Inflammatory Cells

To determine whether there was a local inflammatory response to the nanoparticles we examined infiltration and activation of inflammatory cells following subretinal injection. Infiltration of inflammatory cells such as polymorphonuclear leukocytes (PMN) is an early and acute response to toxic stimuli in the eye [22], [23]. It is likely that if there was a local inflammatory response to the nanoparticles, it would be most pronounced near the injection site. Indeed identifying the precise injection site is quite difficult. The standard transvitreal subretinal injection procedure will create a localized detached region near the injection site in the central-temporal region for the right eye and the central-nasal region for the left eye. However, because detachment can also be caused by histological processing, the detached region cannot be definitively marked as the injection site. In our analysis we evaluated sections taken throughout the entire eye and examined areas both near and far from the injection site.

**Figure 2:**
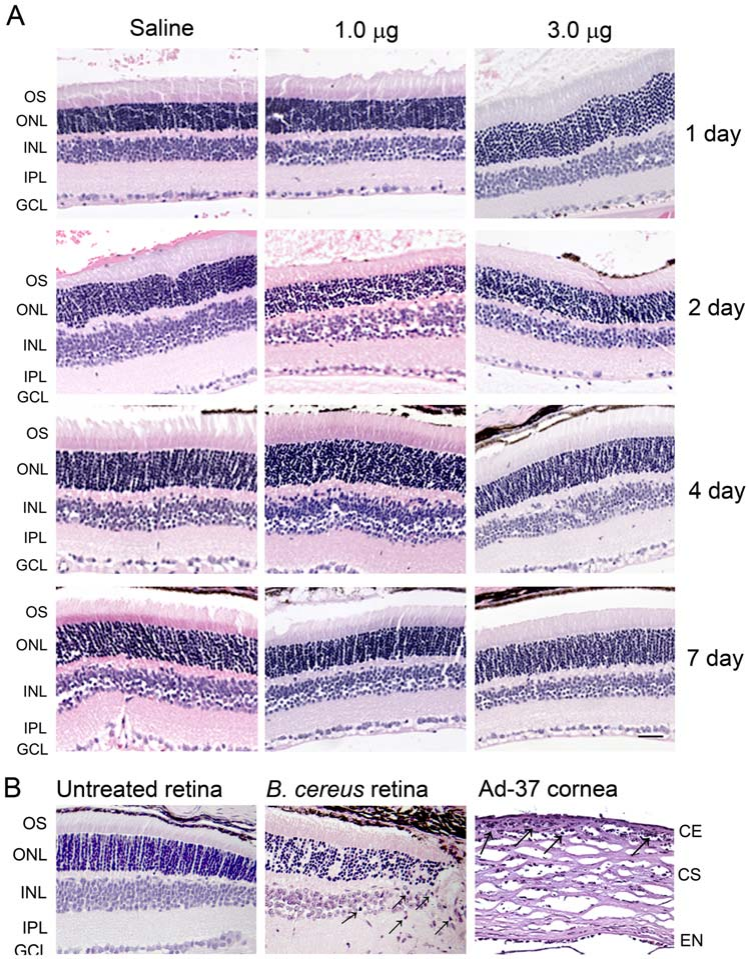
Histological examination of inflammatory cell infiltration in mouse retinas following subretinal delivery of compacted DNA nanoparticles or saline. *A.* Shown are representative images of H&E stained retinal sections of eyes injected with nanoparticles (1.0 and 3.0 µg) or saline at 1, 2, 4 and 7 days PI. No infiltration of inflammatory cells was detected in the injected retinas. *B.* Shown are representative images of control assays. Infiltrating cells were detected in retinal sections of *Bacillus cereus* endophthalmitis eyes (middle panel, shown by arrows) and in corneal sections of Ad37-infected eyes (right panel, shown by arrows). No infiltration was detected on retinal section of untreated eyes (left panel). OS, outer segment; ONL, outer nuclear layer; INL, inner nuclear layer; IPL, inner plexiform layer; GCL, ganglion cell layer; CE, corneal epithelium; CS, corneal stroma; EN, corneal endothelium. Scale bar, 50 µm.

We examined the cross-sections of eyes that had been injected with nanoparticles or saline and performed hematoxylin and eosin (H&E) staining. From these evaluations we did not find clear infiltration of PMN or other inflammatory cells in the injected eyes. Figure 2A are representative images of H&E stained retinal sections showing areas near the injection site. Lower magnification images of the same areas are shown in Figure S1. In these assays, murine eyes with experimentally induced *Bacillus cereus* endophthalmitis [24], [25] and murine eyes with experimentally induced corneal keratitis (by adenovirus type 37, Ad-37) [26] were included as positive controls for infiltration of PMN. As shown in Figure 2B, infiltrating cells were detected in retinal section of *Bacillus cereus* endophthalmitis eyes (middle panel) and in corneal section of Ad37-infected eyes (right panel). No infiltration was detected on retinal sections (left panel) of untreated eyes.

**Figure 3:**
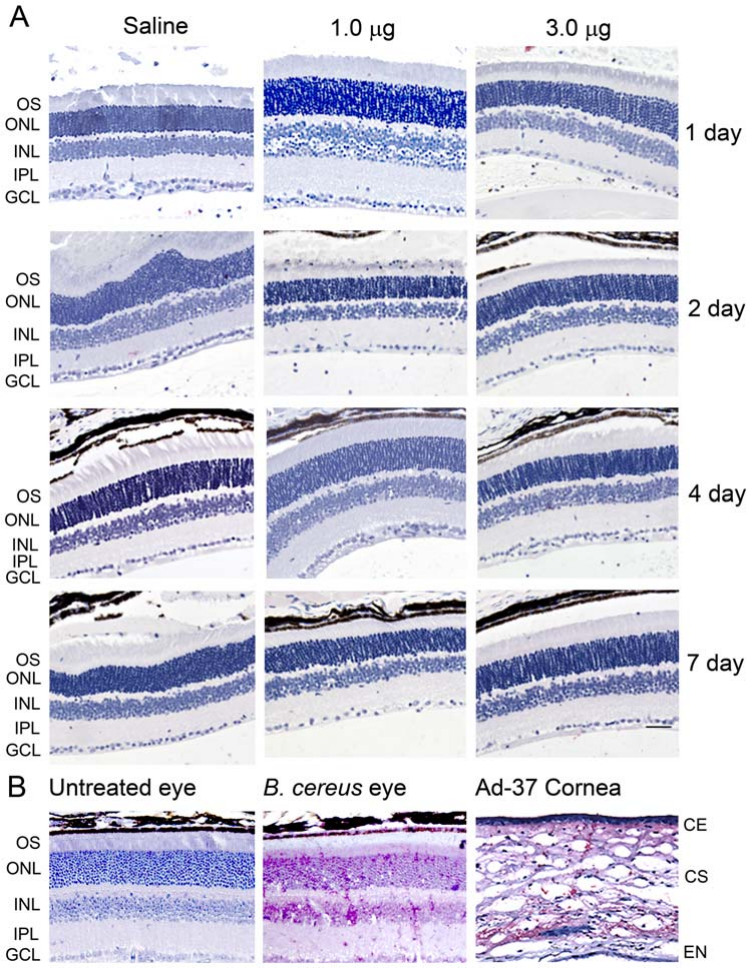
Immunohistochemical examination of MPO expression in mouse retinas following subretinal delivery of compacted DNA nanoparticles or saline. *A.* Shown are representative images of immunohistochemical labeling of MPO on retinal sections of eyes injected with nanoparticles (1.0 and 3.0 µg) or saline at 1, 2, 4 and 7 days PI. No MPO positive labeling was detected in these retinas. *B.* Shown are representative images of control assays. MPO immunoreactivity was detected in the *Bacillus cereus* endophthalmitis eyes (middle panel, *B. cereus* eye) and in the mouse inflammatory corneal sections (right panel, Ad37 cornea). OS, outer segment; ONL, outer nuclear layer; INL, inner nuclear layer; GCL, ganglion cell layer; CE, corneal epithelium; CS, corneal stroma; EN, corneal endothelium. Scale bar, 50 µm.

Subsequently, we examined levels of the myeloid marker myeloperoxidase (MPO) in the retinas of injected eyes. MPO is the most abundant component of azurophilic granules in neutrophils [27] and is also found in the lysosomes of monocytes, PMN leukocytes, and macrophages [28]. During stimulation, MPO is secreted by these cells and activates cellular inflammatory signaling cascades [29]. If there is a local inflammatory response and PMN infiltration following subretinal delivery of nanoparticles, MPO will be activated and released. Thus we examined MPO distribution in the retina by immunohistochemistry following nanoparticle treatment and we found no MPO immunoreactivity in the nanoparticle injected retinal sections. Figure 3A are representative images of the immunohistochemical labeling showing areas near the injection site. Lower magnification images of the same areas are shown in Figure S2. MPO immunoreactivity was markedly elevated in the positive controls (Figure 3B), but there was no detectable MPO signal in either saline or nanoparticle dosed retinas.

**Figure 4:**
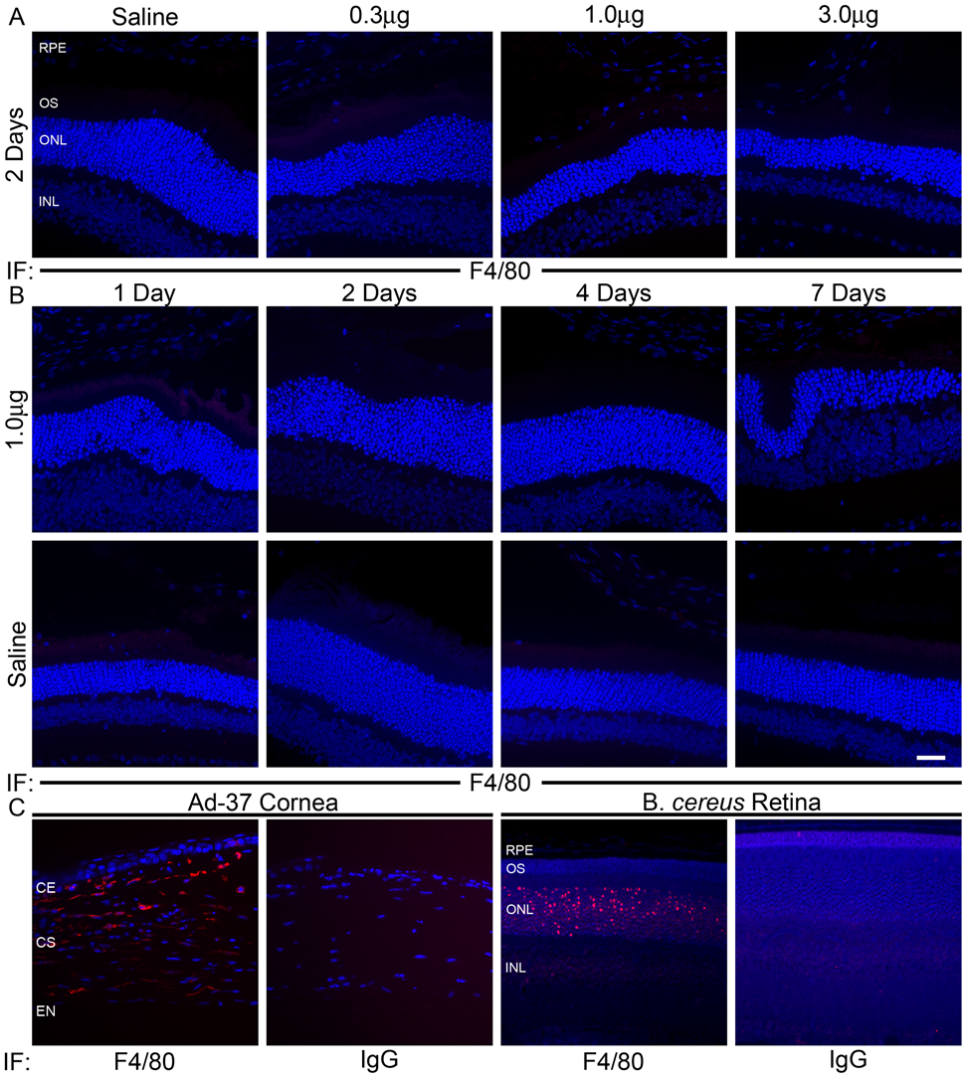
Immunofluorescence examination of F4/80 expression in mouse retinas following subretinal delivery of compacted DNA nanoparticles or saline. *A–B.* Shown are representative images of immunofluorescent examination of F4/80 on retinal sections of eyes that were injected with nanoparticles at (0.3, 1.0 and 3.0 µg) or saline at PI-2 (*A*); or with 1.0 µg nanoparticle at 1, 2, 4 and 7 days PI (*B*). No F4/80 positive labeling was detected in these retinal sections. *C.* F4/80 immunoreactivity was detected in the *Bacillus cereus* endophthalmitis eyes (B. *cereus* retina) and in the mouse inflammatory corneal sections (Ad-37 cornea). RPE, retinal pigment epithelium; OS, outer segment; ONL, outer nuclear layer; INL, inner nuclear layer; CE, corneal epithelium; CS, corneal stroma; EN, corneal endothelium. Scale bar, 40 µm.

We also examined expression of the microglia/macrophage marker F4/80 [30], [31] in retinas that had been injected with nanoparticles. Induction of F4/80 in response to ischemia-induced retinopathy has been described in mouse retina [32]. F4/80 has also been implicated in experimental choroidal neovascularization [33]. Immunofluorescence labeling was performed to determine F4/80 distribution in the retina. As shown in Figure 4A–B, no F4/80 immunoreactivity was detected in nanoparticle or saline injected retinas at any time point or dose. Lower magnification images of the same areas are shown in Figure S3. In contrast, a robust expression of F4/80 was detected in the positive controls (Figure 4C). The absence of microglia/macrophage activation (Figure 4) combined with the observed lack of PMN infiltration (Figure 2) and MPO activation (Figure 3) suggests that there is no local inflammatory response to the nanoparticles.

### Expression of Inflammatory Chemokines

The eye is immune-active and prone to environmental alterations including infection [26], oxygen-induced retinopathy [34], ischemic injury [35] and neovascularization [36]. Interleukin-8 (IL-8/KC), monocyte chemotactic protein-1 (MCP-1) and tumor necrosis factor alpha (TNF-α) are the pro-inflammatory chemokines known to be involved in the ocular inflammatory response [35], [36]. We therefore examined expression of these chemokines in the retinas of eyes that had been injected with DNA nanoparticles.

**Figure 5:**
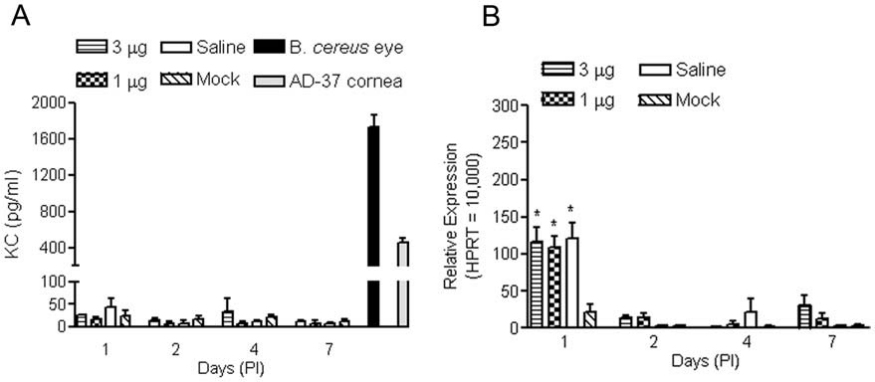
Examination of KC expression in mouse retinas following subretinal delivery of compacted DNA nanoparticles or saline. ELISA (*A*) and qRT-PCR (*B*) were performed to determine KC expression in the retinas of eyes that had been subjected to nanoparticles, saline or mock injection. Assays were performed at 1, 2, 4 and 7 days PI. Ad-37 cornea and *Bacillus cereus* eye samples were included as positive controls in the ELISA. No elevation of KC protein was detected in the injected retinas. A significant elevation of KC was detected in the *Bacillus cereus* endophthalmitis eyes (B. *cereus* eye) and in the inflammatory corneal samples (Ad-37 cornea). KC mRNA levels were significantly elevated in both nanoparticle- and saline-injected retinas at PI-1, compared to the level in the mock injected samples. KC mRNA levels returned to baseline at PI-2. The values shown represent means±SEM of assays from 3–5 injected mice. * P<0.05, compared to mock injected.

KC is a potent chemo-attractant and neutrophil activator and is primarily involved in the initiation and amplification of acute inflammatory reaction processes. It can be produced in response to inflammatory stimuli by a variety of cells types, including macrophages, neutrophils, epithelial cells, and endothelial cells. Since induction of KC in retinas has been observed in response to a variety of local environmental alterations [34], [37], we examined expression of KC in the retinas of eyes that had been injected. Using ELISA, we detected no elevation of KC levels in retinas of saline- or nanoparticle-injected eyes (Figure 5A). As before, the *Bacillus cereus* endophthalmitis eyes and the inflammatory cornea samples were included as positive controls. A striking elevation of KC was detected in these samples (Figure 5A). To confirm our ELISA results, we examined KC mRNA levels in the injected eyes by quantitative real-time (qRT)-PCR. We detected a transient elevation of KC mRNA level in retinas of injected eyes at PI-1. As shown in Figure 5B, KC mRNA levels were increased 3–4 fold in eyes that had been injected with either nanoparticles or with saline at PI-1, but returned to control level at PI-2 (Figure 5B). The concurrent elevation of KC levels in saline and nanoparticle injected eyes suggests that the increase was related to the sub-retinal injection procedure and was not a toxic response to the nanoparticles.

**Figure 6:**
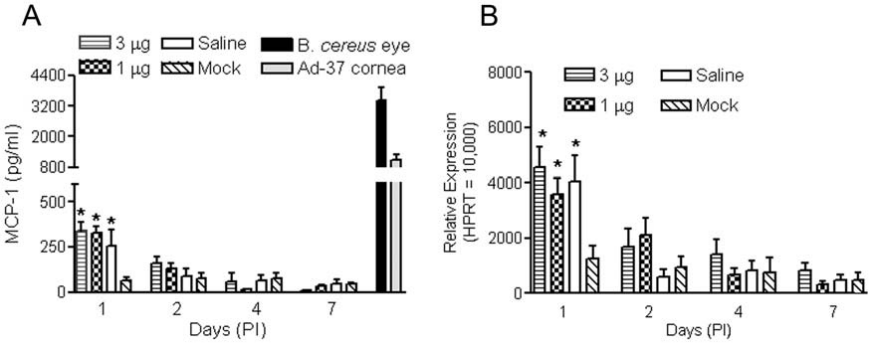
Examination of MCP-1 expression in mouse retinas following subretinal delivery of compacted DNA nanoparticles or saline. ELISA (*A*) and qRT-PCR (*B*) were performed to determine expression of MCP-1 in the retinas of eyes that underwent nanoparticle, saline, or mock injection. Assays were performed at 1, 2, 4 and 7 days PI. Ad-37 cornea and *Bacillus cereus* eye samples were included as positive controls in the ELISA. Levels of MCP-1 protein and mRNA were significantly elevated at PI-1 in both nanoparticle- and saline-injected retinas, compared to the mock injected samples, but returned to the control value at PI-2. A significant elevation of MCP-1 was detected in the *Bacillus cereus* endophthalmitis eyes (B. *cereus* eye) and in the inflammatory corneal samples (Ad-37 cornea). The values shown represent the means±SEM of assays from 3–5 injected mice. * P<0.05, compared to mock injected.

MCP-1 is a member of the small inducible gene family and a member of the chemokine family. It is produced by a variety of cells, including monocytes/macrophages, fibroblasts, and epithelial and endothelial cells. It plays a role in the recruitment of monocytes to sites of injury or infection and is a potent monocyte agonist [36]. Induction of MCP-1 in retinas has been observed in animal models of ischemia reperfusion [35] and has been shown to mediate experimental retinal detachment-induced photoreceptor apoptosis [38]. We therefore examined expression of MCP-1 in the injected retinas by using ELISA and qRT-PCR. A transient increase of MCP-1 protein and mRNA at PI-1 was detected in the retinas following subretinal injection. As shown in Figure 6, the levels of MCP-1 protein or mRNA in nanoparticle- or saline-injected eyes were not different from each other but were about 3–4 fold higher than that in the mock injected eyes. The levels of MCP-1 protein or mRNA returned to control levels by PI-2 (Figure 6). As shown in Figure 6A, markedly elevated levels of MCP-1 were detected in the *Bacillus cereus* endophthalmitis eyes and in the inflammatory cornea samples.

**Figure 7:**
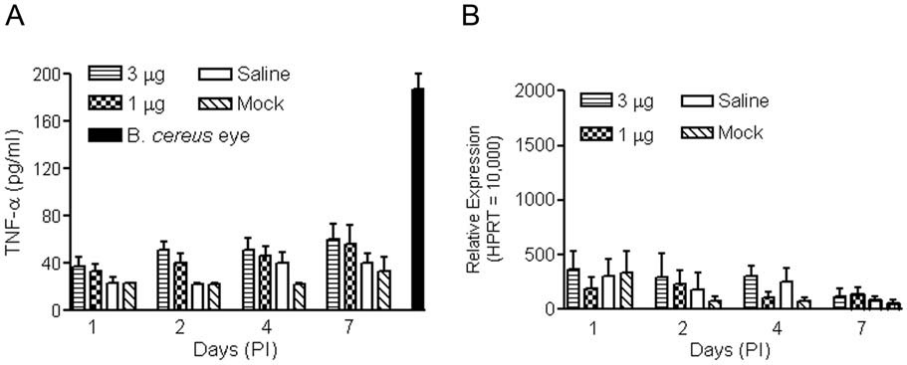
Examination of TNF-α expression in mouse retinas following subretinal delivery of compacted DNA nanoparticles or saline. ELISA (*A*) and qRT-PCR (*B*) were performed to determine expression of TNF-α in the retinas of injected eyes. Assays were performed at 1, 2, 4 and 7 days PI. No significant elevation of TNF-α protein or mRNA was detected in the injected retinas. A significant elevation of TNF-α detected by ELISA was shown in the *Bacillus cereus* endophthalmitis eyes (B. *cereus* eye). The values shown represent the means±SEM of assays from 3–5 injected mice.

TNF-α is produced by activated macrophages/monocytes, and is involved in the acute phase inflammatory reaction. TNF-α is also involved in other types of pathophysiological activities including apoptotic cell death, cellular proliferation, differentiation, and tumorogenesis. It has been reported that TNF-α is expressed and up-regulated in human retinas with proliferative retinopathy [39], [40]. Along with MCP-1, induction of TNF-α has also been shown to mediate experimental retinal detachment-induced photoreceptor apoptosis [38]. Hence we examined expression of TNF-α in retinas of injected eyes. As shown in Figure 7A, no significant elevation of TNF-α was detected in retinas following subretinal delivery of nanoparticles. In contrast, TNF-α level was significantly increased in the experimental *Bacillus cereus* endophthalmitis eyes. qRT-PCR analysis showed that no significant elevation of TNF-α mRNA was detected in the nanoparticle or saline injected eyes (Figure 7B). Protein and mRNA levels of all the chemokines tested in the mock injected eyes were not different from those in the untouched control eyes (data for uninjected control eyes are not shown).

## Discussion

Safety and toxicity testing is a critical component of the development of clinically viable treatments. In this study we show that the CK30PEG-compacted DNA nanoparticles containing a CMV-EGFP expression vector are non-toxic and well-tolerated in the murine eye after subretinal injection. We report no infiltration of PMNs, macrophages, or other inflammatory cells after nanoparticle injection. We also report no nanoparticle-associated alterations in chemokine expression. Transient, early-onset elevations in MCP-1 and KC were detected in nanoparticle and saline injected eyes, and levels of these chemokines quickly returned to normal. Hence, our data indicate that this transient upregulation is likely due to the injection procedure and not the nanoparticle itself.

The eye is an attractive target for gene therapy because of its accessibility, its immune privilege, and numerous genetic disorders. A number of non-viral methods, including electroporation of selected genes [41], injection of antisense oligonucleotides [42] and direct topical delivery (eye drops) using the poly(ethylene oxide)-poly(propylene oxide)-poly(ethylene oxide) (PEO-PPO-PEO) block copolymers [43], have been tested for management of a variety of eye diseases from cornea complications, to diabetic retinopathy and retinal degenerations [42], [44], [45]. Previous promising results have come from delivery of CK30PEG compacted DNA nanoparticles to the subretinal space [14], [21]. We have shown it to be an effective strategy to rescue retinal disease models and are economically and practically advantageous. Here we take the next step and demonstrate that these nanoparticles are also safe and well-tolerated. We detect no significant local inflammatory response or toxicity; information essential and required before any clinic trials can be proposed. This information is doubly important given recent evidence suggesting that some types of nanoparticles do cause a cytotoxic response [46], [47], [48]. Related work studying the safety of non-viral vector gene delivery suggests that cytotoxic effects are dependent on the type of vector, the DNA/vector ratio, and the type of transfected cell [49], suggesting that each type of particle may need to be tested independently. This work is the first study to characterize the local toxic and inflammatory response of retinal tissues to the CK30PEG compacted DNA nanoparticles.

The CK30PEG compacted DNA nanoparticles have been shown to be effective in delivering transgenes to multiple tissues of the lung, brain and eye, and can drive robust expression in both dividing and post-mitotic cells [14], [15], [16], [17], [19], [20]. The nanoparticles are stable in saline and serum, and have been shown to enter cells by a non-traditional mechanism involving specific binding to cell surface nucleolin followed by direct trafficking to the nucleus [50]. A phase I/II clinical trial shows that CK30PEG nanoparticles containing a CFTR vector partially correct CFTR function in CF subjects after intranasal delivery [19]. The safety of airway delivery of these nanoparticles is well documented both in mouse models and in this clinical trial [18], [19]. Following administration of DNA nanoparticles to murine airways, there were no systemic alterations in serum chemistry, hematologic parameters, serum complement levels, IL-6 levels, MIP-2 levels, or in the activity, growth, and grooming of the mice [18]. Only at very high doses (100 µg DNA) was there a modest increase in bronchoalveolar (BAL) neutrophils, a modest increase in BAL IL-6 and KC levels, and a trace to 1+ infiltrate of mononuclear cells surrounding pulmonary veins. The double-blind, dose escalation clinical trial indicated no clinical or laboratory evidence of nanoparticle toxicity [19]. No elevations of IL-6, IL-8, complement, or C-reactive protein in serum or in nasal washings were detected in patients receiving the nanoparticles [19]. These results indicate that CK30PEG nanoparticles encoding CFTR are a promising candidate for CF therapy.

Ocular delivery of CK30PEG DNA nanoparticles has been shown to direct efficient transgene expression in various ocular tissues depending on the route of injection [14]. However, measuring the local inflammatory response to such administration remained a critical step for the further development of these nanoparticles as an ocular treatment strategy. This work investigated the toxic and inflammatory response of retinal tissue to CK30PEG DNA nanoparticles following subretinal delivery. We did not detect any infiltration of inflammatory cells by histological examination or by examination of macrophage/myeloid markers. Up-regulation of the chemokines KC, MCP-1, and TNF-α in eye tissues has been documented in a variety of pathological conditions in the eyes. However, when we examined the protein levels of these inflammatory chemokines we detected only a transient elevation of MCP-1. Importantly, this elevation was detected in eyes that had been injected with either nanoparticles or saline and is thus likely related to the subretinal injection procedure rather than nanoparticle-associated toxicity. MCP-1 in the retina may come from RPE cells [51], [52] as well as infiltrated inflammatory cells. As we did not detect clear cell infiltration, MCP-1 detected in the retina was likely released from RPE cells. We did observe a transient elevation of KC mRNA but not protein at PI-1 in both nanoparticle- and saline-injected retinas. This may reflect a more sensitive detection of mRNA by qRT-PCR than ELISA for this molecule. We also cannot exclude the possibility that the protein level was not altered even though there was an elevation of mRNA. Our current observations on the lack of overt nanoparticle-associated toxicity are in keeping with our previous work which demonstrated that saline and nanoparticle injected eyes had no differences in maximum ERG amplitude (scotopic or photopic) and that after recovery from the subretinal injection procedure the ERG recordings were not different from uninjected control eyes [14].

In summary, this work evaluated the local inflammatory and cellular toxic response to subretinal delivery of compacted DNA nanoparticles. We found no infiltration of inflammatory cells and only a mild and transitory increase in MCP-1 protein that was due to the subretinal injection procedure. Combined with our previous findings, results of this study indicate that subretinal delivery of DNA nanoparticles is a safe and non-toxic approach for ocular gene therapy. This favorable safety profile confirms that compacted DNA nanoparticles can be developed as a clinically relevant therapeutic strategy for the treatment of inherited retinal diseases.

## Materials and Methods

### Plasmid and Nanoparticle Formulation

pZEOGFP5.1 plasmid encoding the EGFP cDNA transcriptionally-controlled by the CMV immediate-early promoter and enhancer [16] was used for the nanoparticle formulation. The nanoparticles were formulated by mixing plasmid DNA with CK_30_PEG10K, a 30-mer lysine peptide with an N-terminal cysteine that is conjugated via a maleimide linkage to 10 kDa polyethylene glycol, as previously described [15]. These nanoparticles consist of a single molecule of DNA per complex [16], have a rod-like morphology with a diameter of 8–11 nm and a length of 150–200 nm [17], are stable in saline for years at 4°C [53], and have a zeta potential of zero [16]. The nanoparticles used in this study were identical in formulation to those previously shown to be capable of driving high levels of gene expression after ocular delivery [14], [21].

### Animals

One-month old C57BL/6 mice (25–30 g) (Charles River Laboratories, Wilmington, MA) were used in this study. All mice studied were maintained in a breeding colony under cyclic light (12-hour light-dark) conditions; cage illumination was approximately 7 foot-candles during the light cycle. All experiments were approved by the local Institutional Animal Care and Use Committees (Oklahoma City, OK, U.S.A.) and conformed to the guidelines on the care and use of animals adopted by the Society for Neuroscience and the Association for Research in Vision and Ophthalmology (Rockville, MD, U.S.A.).

### Subretinal Injection

Transvitreal subretinal injections were performed as described by Nour et al. [54]. The operations were performed under a Carl Zeiss OPMI VISU 140 surgical operating microscope (Thornwood, NY). After anesthesia and complete dilation was achieved, a drop of 2.5% methylcellulose was added to the corneal surface to visualize the fundus. A 28-gauge beveled hypodermic needle (BD Biosciences, Franklin Lakes, NJ) was used to puncture the cornea carefully, avoiding any contact with the lens. The transvitreal subretinal injections were performed with a 33-gauge blunt needle (Hamilton Co., Reno, NV) using a NanoFil microsyringe injector system (World Precision Instruments, Sarasota, FL). 1.0 µL of saline or saline containing DNA nanoparticles at 0.3, 1.0 and 3.0 µg/µL was injected into the subretinal space. These doses have been shown to induce expression of transgenes in ocular tissues [14]. Three to five animals were included in each injection group. A group of animals were anesthetized and underwent corneal puncture without subretinal injection (mock injection control). Retinas or eyes were collected at 1, 2, 4 and 7 days post-injection (PI).

### Histopathology, Immunohistochemistry and Immunofluorescence

Mouse eyes were enucleated and fixed with 4% formaldehyde (Polysciences, Inc., Warrington, PA) in 0.1 M sodium phosphate buffer, pH 7.4 for 16 h at 4°C. After 30 minutes of initial fixation, a small incision was made on the cornea for better fixative penetration. The tissues were then dehydrated through a graded ethanol series and embedded in paraffin. Sections (5 µm thick) were cut along the horizontal meridian throughout the eye, passed through the optic nerve, and were mounted on positively charged slides before being air dried overnight. H&E staining was performed.

Immunohistochemistry was performed as described by Chintakuntlawar et al. [26]. Briefly, retinal sections were treated with 0.01 M citrate buffer (Biopath, Oklahoma City, OK) for epitope retrieval to facilitate antibody recognition. Nonspecific binding was blocked using protein block (Dako, Carpinteria, CA) supplemented with Fc block (CD16/CD32; BD-Pharmingen, San Diego, CA) and 5% rat serum (Jackson ImmunoResearch Laboratories, West Grove, PA). Slides were then incubated with a rabbit polyclonal antibody against myeloperoxidase (MPO) (1∶200) (Neomarkers, Fremont, CA) followed by incubation with biotinylated-polyclonal goat anti-rabbit IgG (1∶500) (Dako, Inc., Carpenteria, CA). Immunodetection was performed with biotin-streptavidin and alkaline phosphatase according to the manufacturer's instructions (Dako, Inc., Carpenteria, CA). The Dako cytomation Liquid Permanent Red (LPR) substrate-chromogen system (Dako, Inc.) was used to develop the APase reaction product. Slides were counterstained with hematoxylin, coverslipped using a synthetic resin, and photographed (Axiovert 135; Carl Zeiss Meditec, Inc.).

Immunofluorescence labeling was performed as described previously [14]. Briefly, after deparaffinization, sections were blocked with PBS containing 5% BSA and 0.5% Triton-X 100 for 1 h at room temperature. Primary antibody incubation with rabbit polyclonal anti-GFP (Molecular Probes Inc. Carlsbad, CA; 1∶250) or with rat monoclonal anti-F4/80 (Serotec, Oxford, UK; 1∶500) was performed at room temperature for 2 h or overnight at 4°C. Following incubation with fluorescence-conjugated secondary antibody (at room temperature for 60 min) and rinses, slides were incubated with DAPI (1∶10,000, Sigma, St. Louis, MO) for 15 minutes to counterstain nuclei, mounted, and cover-slipped. Fluorescent signals were visualized and images were captured using an Olympus AX70 fluorescence microscope (Olympus Corp., Center Valley, PA) with the QCapture imaging software (QImaging Corp., Surrey, BC, Canada).

### ELISA

Sandwich ELISA kits which test for the chemokine MCP-1, mouse KC, and mouse TNF-α were obtained from R&D Systems Inc. (Minneapolis, MN). Assays were performed as recommended by the manufacturer, read using a microplate reader (Molecular Devices, Sunnyvale, CA), and analyzed using SOFTmax software (Molecular Devices, Sunnyvale, CA). Briefly, retinas were homogenized on ice in PBS containing 10 ug/mL leupeptin, 1 mM phenylmethylsulfonyl (PMSF) and 1 ug/mL aprotinin (Sigma-Aldrich, St. Louis, MO). Lysates were centrifuged at 10,000 g for 10 minutes. Supernatants were used undiluted for ELISA experiments. Each sample and the standards provided were analyzed in duplicate, and each time point/treatment was repeated in three to five independent experiments.

### RNA Isolation and Reverse Transcription

Total RNA was isolated from mouse retinas, three to four individual retinas per condition, using Trizol reagent as per the manufacturer's instructions (Invitrogen, Carlsbad, CA). DNAse treatment (Invitrogen) was performed to prevent genomic/nanoparticle DNA contamination and the concentration of RNA was determined using spectrophotometry. Two micrograms of total RNA was reverse transcribed using an oligo-dT primer and SuperScript III reverse transcriptase (Invitrogen) according to instructions provided by the manufacturer.

### Quantitative Real -Time PCR

PCR Universal Master Mix and primer mixes containing the primers and probes for mouse chemokines MCP-1, KC, TNF-α and the internal control gene hypoxanthine guanine phosphoribosyl transferase 1 (HPRT-1) were obtained from Applied Biosystems Inc. (Foster City, CA) and used according to the manufacturer's recommendation. qRT-PCR amplification was performed using a Bio-Rad i-cycler real-time PCR machine (Bio-Rad, Hercules, CA). Each set of primers bridged exons to ensure amplification of cDNA only. Chemokines were tested and normalized against HPRT-1. Normalization was done using the formula 10/2^ΔcT^ where ΔcT  =  cT (gene of interest) – cT (HPRT-1) as described previously [55].

### Inflammatory Eye Samples Used As Positive Controls in This Study

Two types of inflammatory eye samples were used in this study as positive controls. The first type was eyes with experimental *Bacillus cereus* endophthalmitis. The endophthalmitis was induced in C57 BL/6 mice by intravitreal injection of 100 colony-forming units (CFUs) of *Bacillus cereus* and eyes were collected 8 h PI. This is a well established experimental endophthalmitis model and elevation of the inflammatory chemokines has been characterized in the various ocular tissues including the retina [22], [24], [25]. These samples were kindly provided by Dr. Michelle Callegan at the Dean A. McGee Eye Institute (Oklahoma City, OK). The second type of positive control was cornea tissue with experimental inflammation induced by injection of adenovirus type 37 (Ad-37). This model of corneal keratitis is also known to be associated with up-regulation of inflammatory chemokines and infiltration of PMNs [26]. The cornea samples were kindly provided by Dr. James Chodosh at the Dean A. McGee Eye Institute (Oklahoma City, OK). These inflammatory eye samples were used in immunohistochemistry, immunofluorescence labeling, and ELISA.

### Statistical Analysis

ELISA and qRT-PCR for chemokine expression were each performed using retinas from 3–5 injected mice. Mean values were compared by one-way analysis of variance (ANOVA) with Bonferroni's post-hoc pair-wise comparisons (GraphPad Prism 4.0; GraphPad, San Diego, CA).

## Supporting Information

Figure S1 Histological examination of inflammatory cell infiltration in mouse retinas following subretinal delivery of compacted DNA nanoparticles or saline. A. Shown are representative images of H&E stained retinal sections of eyes injected with nanoparticles (1.0 and 3.0 µg) or saline at 1, 2, 4 and 7 days PI. No infiltration of inflammatory cells was detected in the injected retinas. B. Shown are representative images of control assays. Infiltrating cells were detected in retinal sections of Bacillus cereus endophthalmitis eyes (middle panel, shown by arrows) and in corneal sections of Ad37-infected eyes (right panel, shown by arrows). No infiltration was detected on retinal section of untreated eyes (left panel). OS, outer segment; ONL, outer nuclear layer; INL, inner nuclear layer; IPL, inner plexiform layer; GCL, ganglion cell layer; CE, corneal epithelium; CS, corneal stroma; EN, corneal endothelium. Scale bar, 100 µm.
(8.19 MB TIF)

Figure S2 Immunohistochemical examination of MPO expression in mouse retinas following subretinal delivery of compacted DNA nanoparticles or saline. A. Shown are representative images of immunohistochemical labeling of MPO on retinal sections of eyes injected with nanoparticles (1.0 and 3.0 µg) or saline at 1, 2, 4 and 7 days PI. No MPO positive labeling was detected in these retinas. B. Shown are representative images of control assays. MPO immunoreactivity was detected in the Bacillus cereus endophthalmitis eyes (B. cereus eye) (middle panel) and in the mouse inflammatory corneal sections (Ad37-cornea) (right panel). OS, outer segment; ONL, outer nuclear layer; INL, inner nuclear layer; GCL, ganglion cell layer; CE, corneal epithelium; CS, corneal stroma; EN, corneal endothelium. Scale bar, 100 µm.
(2.26 MB TIF)

Figure S3 Immunofluorescence examination of F4/80 expression in mouse retinas following subretinal delivery of compacted DNA nanoparticles or saline. Shown are representative images of immunofluorescent examination of F4/80 on retinal sections of eyes that were injected with nanoparticles at (0.3, 1.0 and 3.0 µg) or saline at PI-2 (A); or with 1.0 µg nanoparticle at 1, 2, 4 and 7 days PI (B). No F4/80 positive labeling was detected in these retinal sections. F4/80 immunoreactivity was detected in the Bacillus cereus endophthalmitis eyes (B. cereus retina) and in the mouse inflammatory corneal sections (Ad-37 cornea) (C). RPE, retinal pigment epithelium; OS, outer segment; ONL, outer nuclear layer; INL, inner nuclear layer; CE, corneal epithelium; CS, corneal stroma; EN, corneal endothelium. Scale bar, 100 µm.
(5.41 MB TIF)
