# Supplementary figures and images for: Ocular Delivery of Compacted DNA-Nanoparticles Does Not Elicit Toxicity in the Mouse Retina
Source: PLoS One. 2009 Oct 12;4(10):e7410. doi: 10.1371/journal.pone.0007410 (PMC2756629; doi:10.1371/journal.pone.0007410)

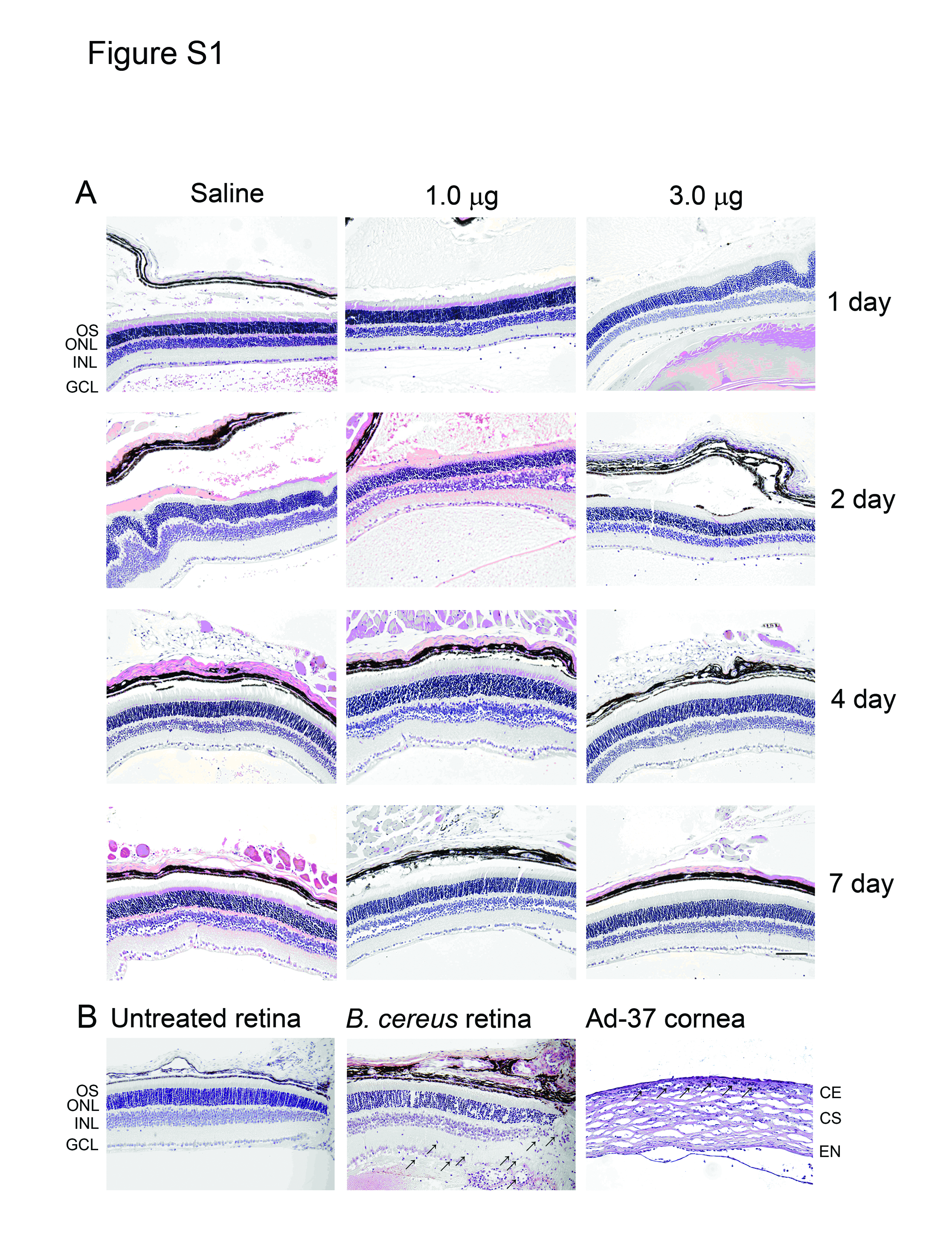

Supplement: Figure S1 — Histological examination of inflammatory cell infiltration in mouse retinas following subretinal delivery of compacted DNA nanoparticles or saline. A. Shown are representative images of H&E stained retinal sections of eyes injected with nanoparticles (1.0 and 3.0 µg) or saline at 1, 2, 4 and 7 days PI. No infiltration of inflammatory cells was detected in the injected retinas. B. Shown are representative images of control assays. Infiltrating cells were detected in retinal sections of Bacillus cereus endophthalmitis eyes (middle panel, shown by arrows) and in corneal sections of Ad37-infected eyes (right panel, shown by arrows). No infiltration was detected on retinal section of untreated eyes (left panel). OS, outer segment; ONL, outer nuclear layer; INL, inner nuclear layer; IPL, inner plexiform layer; GCL, ganglion cell layer; CE, corneal epithelium; CS, corneal stroma; EN, corneal endothelium. Scale bar, 100 µm. (8.19 MB TIF) [file pone.0007410.s001.tif]

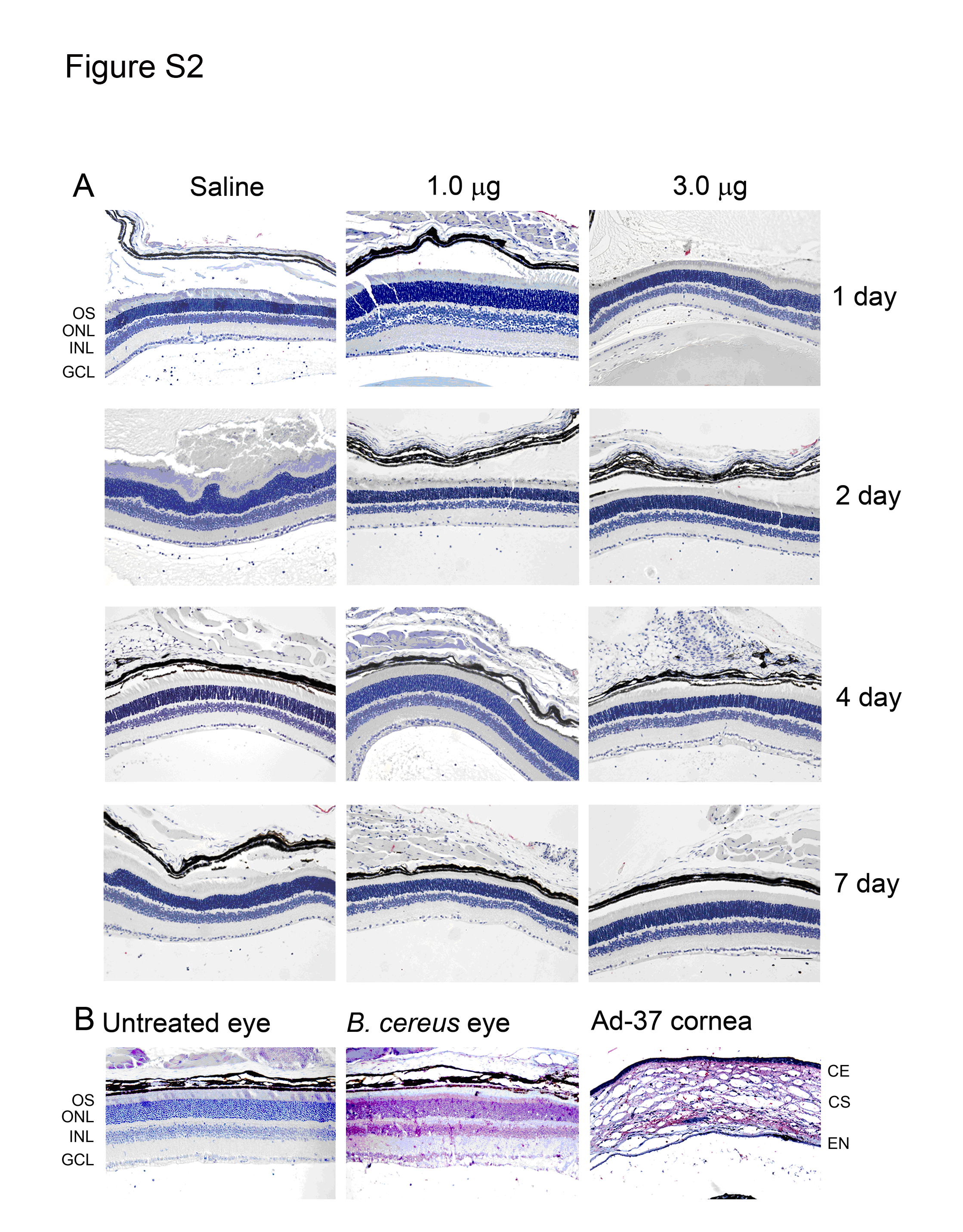

Supplement: Figure S2 — Immunohistochemical examination of MPO expression in mouse retinas following subretinal delivery of compacted DNA nanoparticles or saline. A. Shown are representative images of immunohistochemical labeling of MPO on retinal sections of eyes injected with nanoparticles (1.0 and 3.0 µg) or saline at 1, 2, 4 and 7 days PI. No MPO positive labeling was detected in these retinas. B. Shown are representative images of control assays. MPO immunoreactivity was detected in the Bacillus cereus endophthalmitis eyes (B. cereus eye) (middle panel) and in the mouse inflammatory corneal sections (Ad37-cornea) (right panel). OS, outer segment; ONL, outer nuclear layer; INL, inner nuclear layer; GCL, ganglion cell layer; CE, corneal epithelium; CS, corneal stroma; EN, corneal endothelium. Scale bar, 100 µm. (2.26 MB TIF) [file pone.0007410.s002.tif]

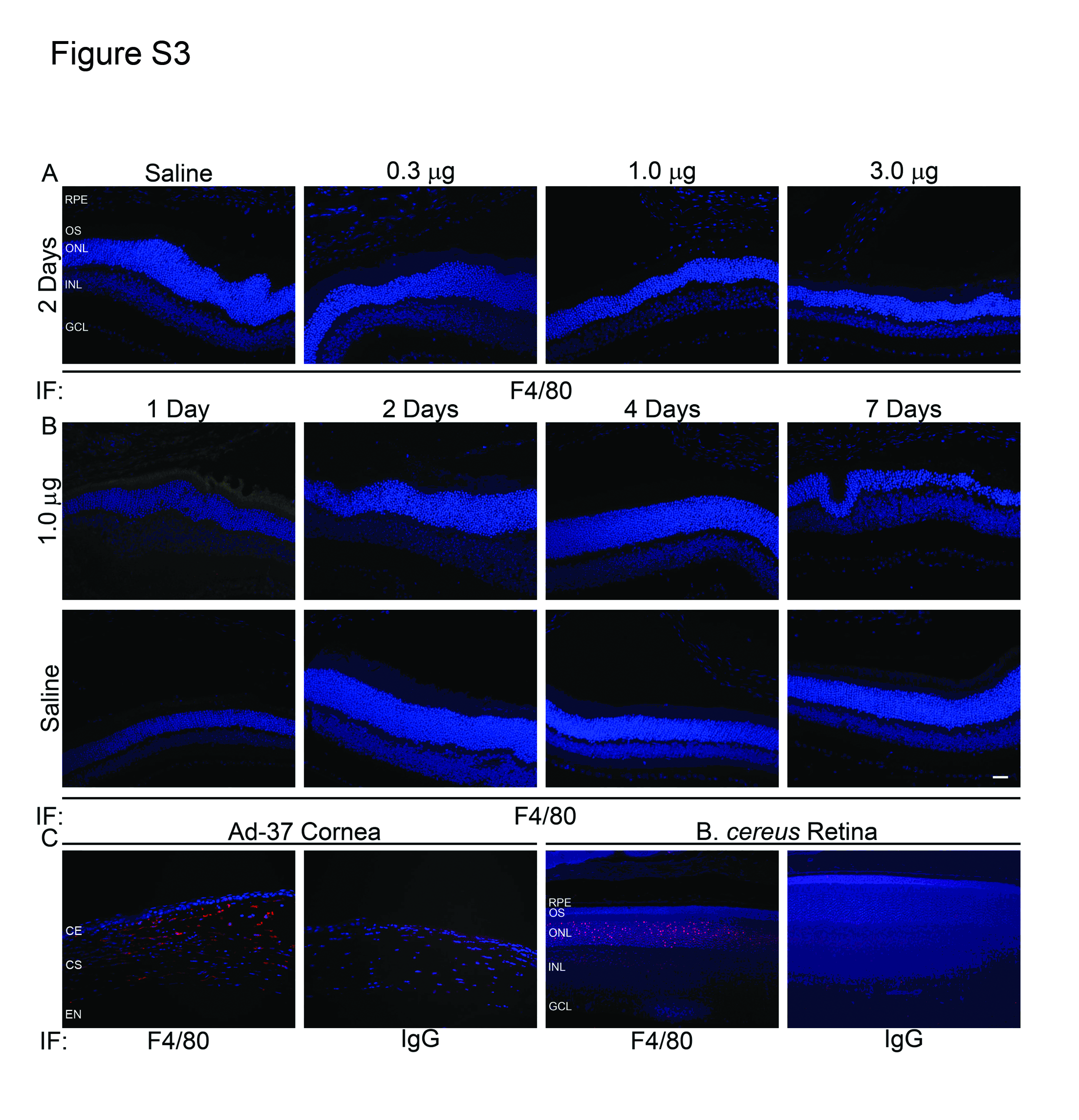

Supplement: Figure S3 — Immunofluorescence examination of F4/80 expression in mouse retinas following subretinal delivery of compacted DNA nanoparticles or saline. Shown are representative images of immunofluorescent examination of F4/80 on retinal sections of eyes that were injected with nanoparticles at (0.3, 1.0 and 3.0 µg) or saline at PI-2 (A); or with 1.0 µg nanoparticle at 1, 2, 4 and 7 days PI (B). No F4/80 positive labeling was detected in these retinal sections. F4/80 immunoreactivity was detected in the Bacillus cereus endophthalmitis eyes (B. cereus retina) and in the mouse inflammatory corneal sections (Ad-37 cornea) (C). RPE, retinal pigment epithelium; OS, outer segment; ONL, outer nuclear layer; INL, inner nuclear layer; CE, corneal epithelium; CS, corneal stroma; EN, corneal endothelium. Scale bar, 100 µm. (5.41 MB TIF) [file pone.0007410.s003.tif]
